# Combination of serology and PCR analysis of environmental samples to assess *Coxiella burnetii* infection status in small ruminant farms

**DOI:** 10.1128/aem.00931-25

**Published:** 2025-08-29

**Authors:** Ana L. García-Pérez, Ion Iñaki Zendoia, Dulce Ferrer, Jesús F. Barandika, Cristina Ramos, Roberto Vera, Tomeu Martí, Antònia Pujol, Aitor Cevidanes, Ana Hurtado

**Affiliations:** 1 Animal Health Department, NEIKER – Basque Institute for Agricultural Research and Development, Bizkaia Science and Technology Park, Basque Research and Technology Alliance (BRTA) 568373 , Bizkaia, Spain; 2 IRFAP - Institut de Recerca i Formació Agroalimentària i Pesquera de les Illes Balears 738257 , Palma, Spain; Centers for Disease Control and Prevention, Atlanta, Georgia, USA

**Keywords:** *Coxiella burnetii*, Q fever, small ruminants, serology, environmental dust, real-time PCR, quantitative PCR

## Abstract

*Coxiella burnetii*, the causative agent of Q fever, accumulates on dust of farm premises with infected animals, but the interpretation of PCR detection on dust is challenging. To investigate whether bacterial load in environmental dust together with the within-flock seropositivity could be indicative of the *C. burnetii* infection status in small-ruminant flocks, 249 farms (202 sheep, 18 goats, and 29 sheep-goat mixed) in the Balearic Islands were investigated. Dust samples were analyzed by real-time PCR targeting *C. burnetii* IS*1111*, and *C. burnetii* loads (genome equivalents/mg dust) were estimated by quantitative real-time PCR amplification of *com1* to categorize the farms into four levels. Sera from 546 yearlings and 1,002 adult females in 79 flocks were tested by ELISA. Despite a widespread distribution of *C. burnetii* in environmental dust, only 6.0% and 16.1% had high (>1,000) or moderate (100–1,000) *C. burnetii* loads, respectively. These same farms had significantly higher within-flock percentage of *C. burnetii* seropositive animals (~35%) than flocks with low loads or *C. burnetii*-negative. These results and the positive correlation observed between within-flock percentage of seropositive animals and *C. burnetii* environmental contamination indicate that dust PCR followed by serological analysis of flocks with high and moderate loads could be used to identify herds potentially infected with *C. burnetii*. SNP genotyping of *Coxiella* PCR-positive dust samples identified SNP-6 as the predominant genotype in small ruminants in the Balearic Islands, along with the sporadic presence of SNP-4—a clearly different genotype distribution than in northern Spain, where the clinical spectrum of human Q fever is clearly different.

**IMPORTANCE:**

The identification of flocks with active *C. burnetii* infection is crucial to implement control measures and prevent human Q fever cases. This study demonstrates the relevance of combining dust PCR with serology to identify *C. burnetii-*infected herds, a strategy that could help to identify the animal source of human Q fever outbreaks and define priority countermeasures. This study also provided, for the first time, an overview of *C. burnetii* infection in sheep and goats in the Balearic Islands and identified the factors associated with higher risk of environmental *C. burnetii* contamination. Strain characterization allowed the identification of the most prevalent *C. burnetii* genotypes in this region of eastern Spain, showing clear differences in genotype distribution with the northern area, which could explain the different clinical spectrum of human Q fever cases in both geographical areas.

## INTRODUCTION

Q fever is a globally distributed zoonosis that sometimes causes human outbreaks that can affect large numbers of people, some of whom may require hospitalization (1). Domestic ruminants are the main reservoirs of the causative agent *Coxiella burnetii* (2, 3), but wildlife cannot be ruled out as a source of infection (4). *Coxiella burnetii* infection of domestic ruminants can cause abortions, stillbirth, premature birth, and weak offspring. In 2023, Spain was the European country that reported the highest incidence of human Q fever (5). The Spanish animal health authorities have established control plans (6) and recommendations, already adapted in some autonomous communities, focused on the control of abortions and the vaccination of small ruminants with an inactivated vaccine in phase I. The vaccine, when applied to previously uninfected animals, significantly reduces the number of abortions and the excretion of the bacteria through different routes: vaginal, fecal, and milk (7). However, vaccination is usually applied when infection is already present in the herd and an outbreak of abortion occurs (8), which hampers infection control, as infected animals can excrete *C. burnetii* during several consecutive parturitions (9).

*Coxiella burnetii* is a highly persistent bacterium in the environment (10, 11), where it can remain viable for years, leading to further reactivations of the infection. After abortion or normal parturition of *C. burnetii-*infected animals, high amounts of bacteria are shed (12, 13), aerosolized, and deposited on dust on various surfaces in animal facilities (14). PCR analysis of environmental dust samples collected soon after abortions or normal parturition can be used to identify farms with recent *C. burnetii* infections (14, 15). However, PCR detection of *C. burnetii* in environmental dust may overestimate the number of infected farms, as *C. burnetii* spore-like small-cell variant (SCV) forms can remain in the environment for years without necessarily being viable (9, 16). It is therefore necessary to complement dust PCR analyses with the analyses of animal samples to check for recent contact of the herd with the infection.

The best way to identify coxiellosis as the cause of the abortions is through direct detection of *C. burnetii* by microscopic examination of placental smears and placental lesions, immunohistochemistry, or detection of high bacterial loads by PCR in placenta or the fetus. PCR detection of *C. burnetii* in vaginal fluids from recently aborted animals can also point towards a putative relationship between abortion and *C. burnetii*, which requires further confirmation (17). The degree of exposure of the herd to *C. burnetii* can also be assessed by detecting antibodies (18), which together with PCR tests can provide information on whether a herd/flock is clinically affected with Q fever (19). For this purpose, ELISA is the most widely used serological technique over complement fixation or indirect immunofluorescence tests (20). However, it is of paramount importance to choose an ELISA kit with a high sensitivity and specificity (21).

Acute Q fever in humans can result in flu-like symptoms, such as fever, headache, fatigue, and myalgia, and can also lead to more severe symptoms, such as pneumonia or hepatitis (22). In Spain, depending on the geographical area, Q fever manifests differently with cases of pneumonia predominating in the north and fever and hepatitis in the center-south and the Canary Islands (23). Although not yet proven, the different clinical manifestations could be related to the animal reservoir species and the different *C. burnetii* genotypes. It is important to identify the genotypes causing coxiellosis in ruminants and compare them with those of isolates recovered from human infections not only for source attribution but also to investigate the genetic determinants associated with the variety of clinical manifestations of the disease. Several techniques have been developed to genotype *C. burnetii*. Multiple-locus variable number tandem repeat analysis (MLVA) (24) and multispacer sequence typing (MST) (25) are highly discriminatory techniques, but they are very laborious. Besides, MLVA is difficult to standardize between laboratories. Alternatively, single nucleotide polymorphism (SNP) genotyping, despite its lower discriminatory power, is fast and easy to interpret, which in practice allows easier comparison of genotypes obtained from animal, environmental, and human samples (26). Previous studies in the Basque Country have shown a wide genetic diversity of *C. burnetii* in domestic ruminants (15), and the predominant genotypes in ruminants (SNP8 and SNP1) have been associated with cases of human Q fever, with pneumonia as the main symptom (27–29).

The present study was conducted in small ruminant farms from the Balearic Islands, a region where a retrospective study carried out between 2003 and 2011 identified 87 cases of human Q fever (30). Pneumonia was diagnosed in 44.8% of patients, a febrile episode in 24.1%, and acute hepatitis in 21.8%. In a more recent study carried out in one of the islands (Majorca) in 2017–2022, the clinical symptoms identified in 223 cases of acute Q fever (102 confirmed and 121 probable) included prolonged febrile syndrome (48.0%), pneumonia (21.9%), acute hepatitis (17.0%), and pericarditis and/or myocarditis (2.6%) (31). However, no studies had been performed before to identify the animal reservoir and the *C. burnetii* genotypes involved. Therefore, in this work, we investigated the presence of *C. burnetii* in environmental samples collected in small ruminant farms, as well as the exposure of the animals to the infection, expressed as presence/absence of antibodies. We hypothesized that within-flock seroprevalence, together with bacterial load in environmental dust, would indicate the status of *C. burnetii* infection. This approach, combining serology with molecular techniques, could be of interest to design strategies to identify infected herds and to design control measures.

## RESULTS

### *Coxiella burnetii* in dust

Six percent (15/249) of small ruminant farms had more than 1,000 genome equivalents (GE) of *C. burnetii* per mg of dust taken inside animal facilities, and 16.0% (40/249) had loads between 100 and 1,000 GE/mg dust (Table 1). Farms with lower loads (<100 GE/mg dust) predominated (50.6%, 126/249), and most of these (84.9%, 107/126) had less than 40 GE/mg dust. Goat and mixed farms showed a higher percentage of high or moderate *C. burnetii* loads in environmental dust (27.8% and 27.6%, respectively) compared to sheep farms (20.8%). However, the difference was not statistically significant (χ² = 1.04; *P* > 0.05). The number of farms negative for the presence of *C. burnetii* DNA or with low *C. burnetii* loads in dust was slightly, but not significantly, higher in sheep (79.2%) than in goats (72.2%) and mixed farms (72.4%).

**TABLE 1:** Distribution of the loads of *C. burnetii* detected in the farm environmental dust according to the type of flock

GE *C. burnetii* / mg of dust	Total *N* (%)	Sheep *N* (%)	Goat *N* (%)	Mixed *N* (%)
>1,000	15 (6.0)	12 (5.9)	1 (5.6)	2 (6.9)
100–1,000	40 (16.0)	30 (14.9)	4 (22.2)	6 (20.7)
<100	126 (50.6)	106 (52.5)	4 (22.2)	16 (55.2)
Negative	68 (27.3)	54 (26.7)	9 (50.0)	5 (17.2)

**Fig 1:**
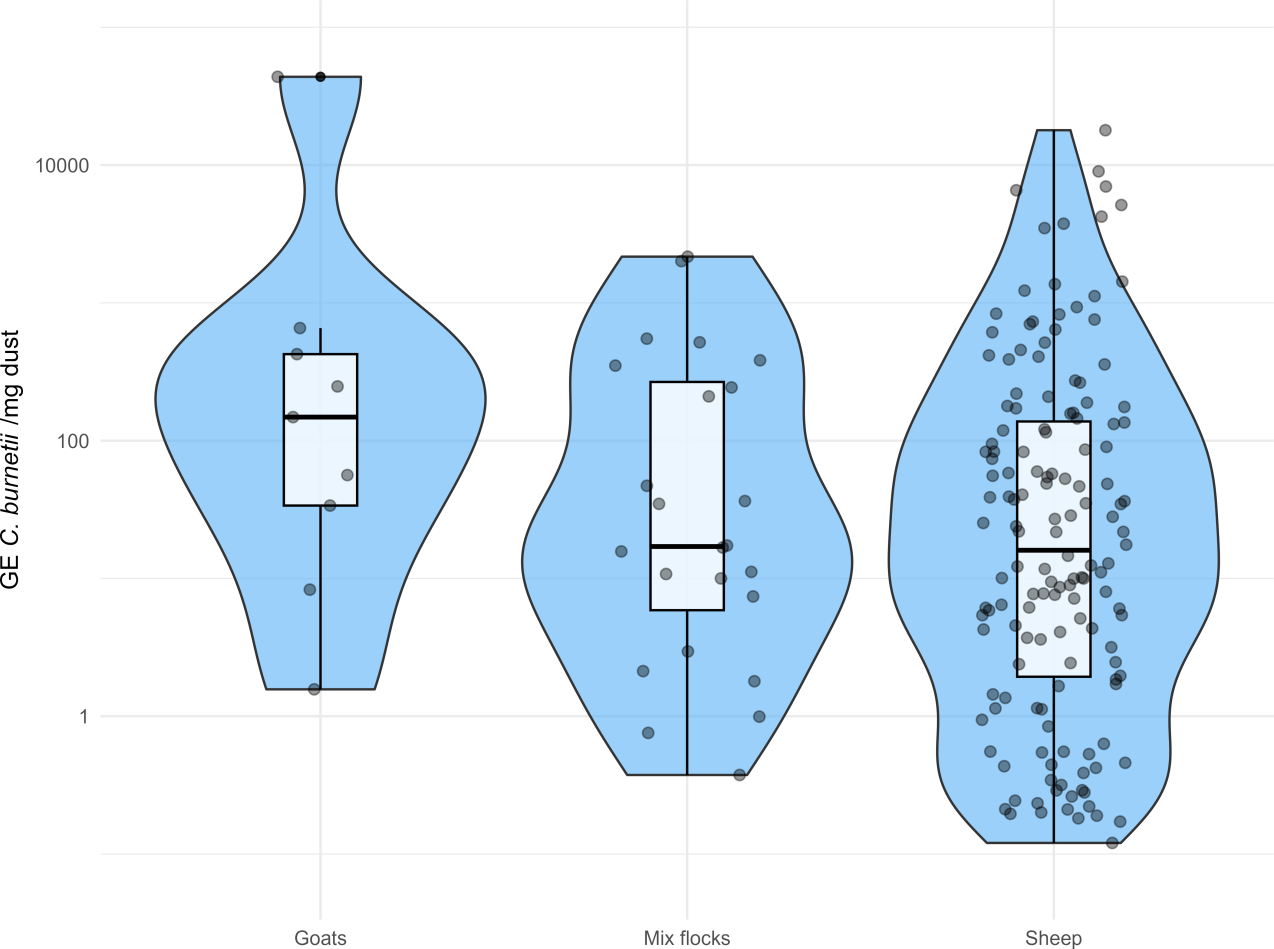
Violin plots showing the distribution of *C. burnetii* bacterial load in positive environmental dust samples (genome equivalents [GE] per milligram of dust) for each flock type. Each violin represents the kernel density estimation of the GE values. Overlaid boxplots display the interquartile range (IQR), with thick black lines indicating the median and the box limits representing the first and third quartiles. Individual data points are shown as jittered dots (light blue) to visualize sample distribution and overlap. The y-axis is presented on a logarithmic scale.

The *C. burnetii* loads estimated by qPCR reached up to 43,700 GE/mg dust, and this maximum value corresponded to a goat farm. The maximum value detected in sheep was 17,911 GE/mg dust and 2,166 GE/mg dust in mixed farms. However, statistical analysis using the Kruskal-Wallis test (KW χ² = 3.81, *P* = 0.14) did not detect statistically significant differences in bacterial loads between flock types (Fig. 1).

### Farm characteristics associated with dust *C. burnetii* positivity

Analysis of the molecular results, together with information obtained from the questionnaires covering 223 farms, showed that PCR detection of *C. burnetii* in dust was associated with farms with a census of more than 100 animals, where operators wore clothing exclusively for use inside animal facilities, and with poorly ventilated barns (Table 2). The presence of wildlife in the environment does not seem to represent a risk, nor does having suffered abortions recently. According to the questionnaires, 12 flocks reported abortions in the period 2018–2022. Seven of them had PCR-negative dust samples and five tested positive. Interestingly, the goat herd with more recent abortions (year 2022) showed a moderate load of *C. burnetii* in the farm environmental dust (657 GE of *C. burnetii*/mg dust), while the other four flocks (one mixed and three ovine flocks), which suffered abortions in 2018 or 2019, revealed low *C. burnetii* loads (<40 GE/mg dust).

**TABLE 2:** Summary of the best model associated with the presence of *C. burnetii* DNA in environmental dust in small ruminant farms resulting from the multivariate logistic regression analysis

	Estimate	Z value	*P* (>|t|)^*a*^	Odds ratio (95% IC)
History of abortions				
No (ref.)	–^*b*^	–	–	–
Yes	−1.028	−1.506	0.132	0.36 (0.09–1.35)
Animal census				
<50 (ref.)	–	–	–	–
50–100	0.120	0.281	0.778	1.13 (0.49–2.60)
>100	1.982	3.520	**<0.001**	7.26 (2.41–21.89)
Exclusive cloth and footwear				
No (ref.)	–	–	–	–
Yes	0.881	2.222	**0.026**	2.67 (1.12–6.35)
Contact with wildlife				
No (ref.)	–	–	–	–
Yes	−1.244	−3.094	**0.002**	0.29 (0.13–0.63)
Ventilation of the barn				
Good (ref.)	–	–	–	–
Poor	0.982	2.223	**0.026**	2.67 (1.17–6.67)

^*a*^ *P* values of <0.05 are considered statistically significant and are represented in bold.

^*b*^ – indicates not applicable.

### *Coxiella burnetii* SNP genotypes

A total of 103 DNA samples were analyzed by SNP genotyping. Complete SNP genotypes were obtained for 65 samples (Table 3) and incomplete or inconclusive genotypes for 38 samples. Two genotypes were identified, with genotype SNP-6 being the most frequent (92.3%, 60/65), while SNP-4 was only detected in five farms (7.7%, 5/65). Genotype SNP-6 was detected on farm premises of the three types of flocks and SNP-4 in sheep and mixed flocks.

**TABLE 3:** Distribution of SNP genotypes according to flock type

Genotype	Sheep (*n* = 49)	Goats (*n* = 4)	Mixed flocks (*n* = 12)
SNP-6	45	4	11
SNP-4	4	0	1

Only in one of the five farms that tested PCR-positive in dust and reported abortions in the period 2018–2022 was *C. burnetii* successfully genotyped. It corresponded to a dust sample collected from a 167-head goat farm where abortions were reported the same year of sampling (2022), and the genotype was identified as SNP-6.

### Relationship between seropositivity and *C. burnetii* DNA loads in the environment

Sera were collected from 48 sheep, 14 goats, and 17 mixed flocks. From each flock, blood samples from a maximum of 20 animals (preferentially 10 yearlings and 10 adult females with more than one parturition) were randomly selected. In 17 flocks, blood samples could not be collected from yearlings. As a result, 1,548 sera were tested serologically by ELISA, 546 sera from yearlings and 1,002 from adult females. The overall percentage of seropositivity was significantly higher in goats than in sheep, both when comparing the seropositivity in single-species flocks (30.7% goats vs. 20.1% sheep) (χ² = 12.09; *P* < 0.05) and when comparing the seropositivity of goats and sheep within mixed flocks (25.2% in goats vs. 15.8% in sheep) (χ² = 4.71; *P* < 0.05) (Table 4). A total of 79.7% of the small-ruminant flocks had at least one seropositive animal (63/79).

**TABLE 4:** ELISA seropositivity at flock and animal species level by type of flock

Flock type	Proportion of seropositivity		
	Flocks	Sheep	Goats
Sheep	39/48 (81.3%)	186/924 (20.1%)	na
Goats	10/14 (71.4%)	na^*a*^	65/212 (30.7%)
Mixed flocks	14/17 (82.4%)	42/265 (15.8%)	37/147 (25.2%)

^*a*^ na, non-applicable.

Overall, within-flock percentage of seropositive animals equal to or higher than 30% was observed in 20 flocks (25.3%, 20/79), with eight of them (10.1%, 8/79) reaching values higher than 50%. Considering the age, a significantly lower overall seropositivity was observed in yearlings (13.7%; 75/546) than in adult females (25.5%; 255/1002) (χ² = 28.90; *P* < 0.001). This age-related pattern was consistently observed across flock types (sheep, goats, and mixed flocks), with the most pronounced difference found in goats: yearlings (7.04%; 5/71) vs. adult females (42.55%; 60/141) (χ² = 28.00; *P* < 0.00001). In 10 of the 62 flocks (16.1%), in which both yearlings and adults were sampled, within-flock percentage of seropositive animals was higher than 30% in both age groups, exceeding 50% in four of these flocks.

Seropositivity results according to the bacterial load in dust and the type of flock are summarized in Table 5. Farms with more than 1,000 *C*. *burnetii* GE/mg dust showed the highest mean within-flock percentage of seropositive animals (35.5%). Those with moderate loads showed a mean percentage of seropositive animals of 32.3%, and those with low loads had a mean percentage of seropositive animals of 14.6%. The mean percentage of seropositive animals in negative flocks was 13.1%.

**TABLE 5:** Percentage of seropositive animals according to *C. burnetii* load in environmental dust and type of flock (*n* = 79)^*a*^

GE *C. burnetii* / mg dust	Type flock	Tested		*N* animals ELISA + (%)
		*N* flocks	*N* animals	
>1,000	Sheep	9	173	51 (29.5)
	Goats	1	23	22 (95.7)
	Mixed	2	54	16 (29.6)
	**Total**	**12**	**250**	**89** (**35.5**)
100–1,000	Sheep	6	123	40 (32.5)
	Goats	3	55	19 (34.5)
	Mixed	4	116	36 (31.0)
	**Total**	**13**	**294**	**95** (**32.3**)
<100	Sheep	20	346	51 (14.7)
	Goats	4	52	13 (25.0)
	Mixed	7	155	17 (11.7)
	**Total**	**31**	**553**	**81** (**14.6**)
Negative	Sheep	13	282	38 (13.5)
	Goats	6	82	11 (13.4)
	Mixed	4	87	10 (11.5)
	**Total**	**23**	**451**	**59** (**13.1**)

^*a*^ Totals─independently of the type of flock─for each category group of *C. burnetii* load (GE/mg) in dust are indicated in bold.

**Fig 2:**
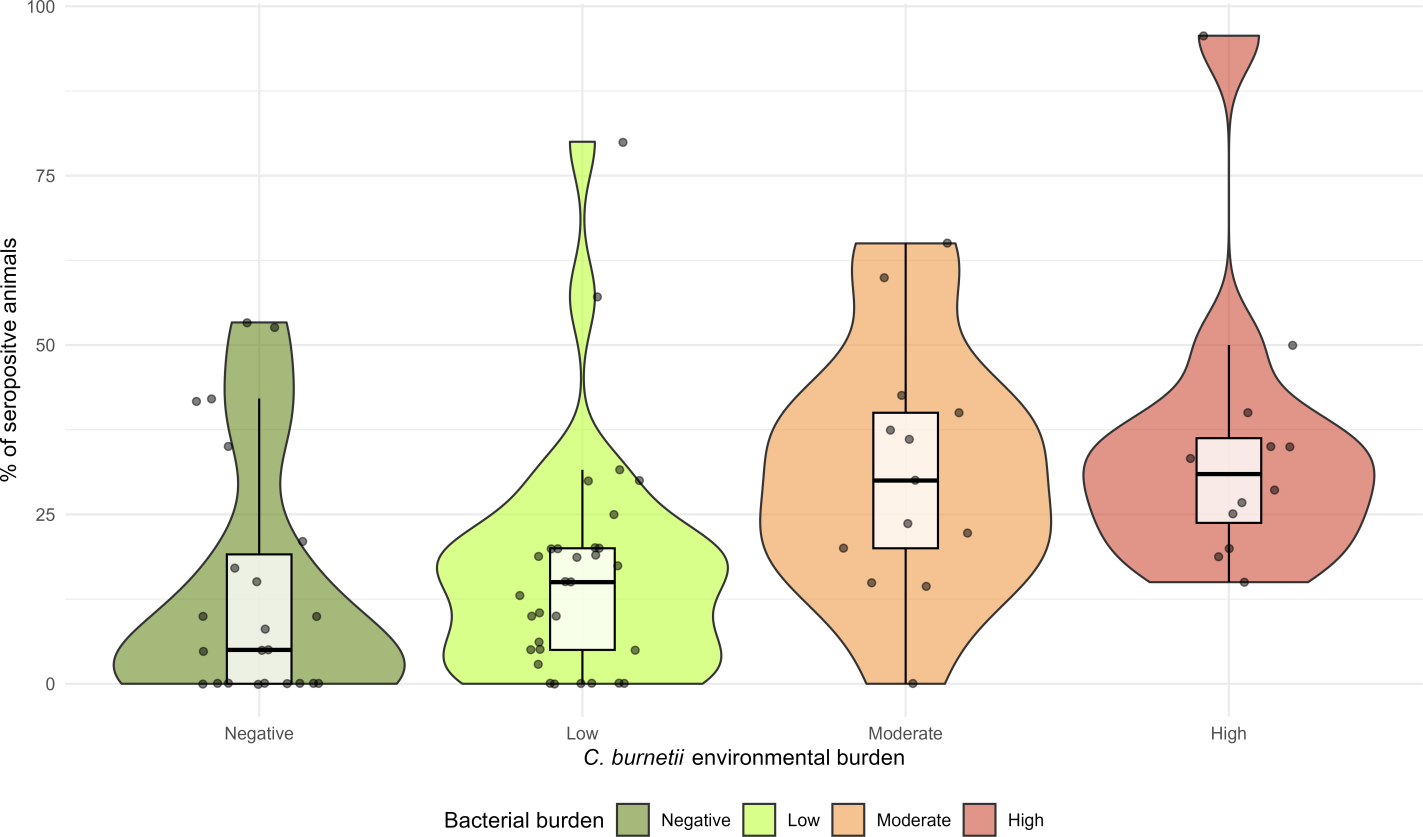
Violin plots representing the within-flock percentage of seropositive animals (%) in relation to *C. burnetii* environmental contamination categories. Each violin represents the kernel density estimation of the percentage of seropositive animal data. Overlaid boxplots display the interquartile range (IQR), with thick black lines indicating the median and the box limits representing the first and third quartiles. Individual data points are shown as jittered dots to visualize sample distribution and overlap.

Kruskal-Wallis tests revealed significant differences in seropositivity across environmental *C. burnetii* load categories (KW χ² = 18.468, *P* < 0.001). The post-hoc pairwise comparison in Dunn’s test demonstrated that on farms with a high *C. burnetii* burden in dust, flocks had significantly higher percentage of seropositive animals compared to those exposed to low (*P* = 0.0177) or negative burdens (*P* = 0.0038). Similarly, farms with a moderate load showed significantly higher values than negative farms (*P* < 0.05). No significant differences were found between high and moderate loads or between low loads and negative farms (*P* > 0.05) (Table 6; Fig. 2).

**TABLE 6:** Pairwise comparison of seropositivity between flocks classified in the different *C. burnetii* environmental load categories

*C. burnetii* loads in dust^*a*^	Z statistic	*P*-value	Adjusted *P*-value	Significance level^*b*^
High–low	2.9721	0.0030	0.0177	*
High–moderate	0.3761	0.7068	1.0000	ns
High–negative	3.4147	0.0006	0.0038	**
Moderate–low	−2.6024	0.0093	0.0555	ns
Moderate–negative	3.0705	0.0021	0.0128	*
Low–negative	0.7468	0.4552	1.0000	ns

^*a*^ High, >1,000 *C*. *burnetii* GE/mg dust. Moderate, 100–1,000 *C. burnetii* GE/mg dust. Low, <100* C. burnetii* GE/mg dust. Negative, not detected in dust by real-time PCR targeting the IS*1111*.

^*b*^ ns, non-significant; *P = 0.05; **P = 0.001.

**Fig 3:**
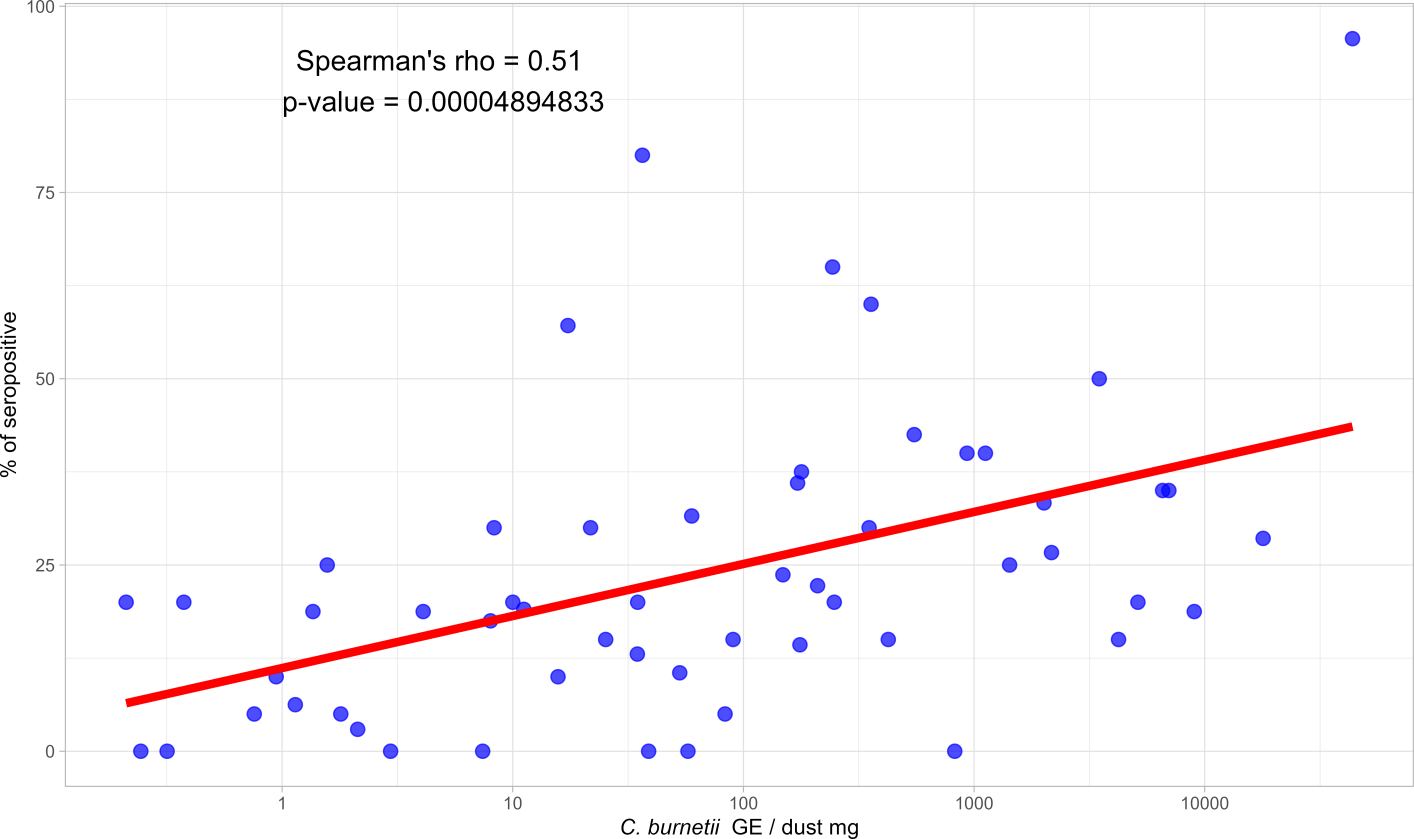
Correlation between the mean within-flock percentage of seropositive animals (%) against *C. burnetii* and the bacterial load in environmental dust (GE/dust mg). The red line indicates a fitted linear trend.

The percentage of seropositive animals was higher in both yearlings and adults in flocks from farms with high or moderate *C. burnetii* environmental loads. The within-flock percentage of seropositive animals in adult females in relation to *C. burnetii* environmental loads revealed significant differences, as shown by the Kruskal-Wallis test (KW χ² =15.195, *P* < 0.05). Post-hoc Dunn’s test indicated that seropositivity was significantly higher in environments with a high bacterial load compared to negative (*P* = 0.005) or low (*P* = 0.044) load. No significant differences in seropositivity were observed between high and moderate loads or between low and moderate loads. In contrast, the Kruskal-Wallis test did not detect statistically significant differences for seropositivity in yearlings according to bacterial loads (KW χ² =7.289, *P* = 0.063). Overall, a positive and significant correlation between within-flock percentage of seropositive animals and environmental contamination by *C. burnetii* was observed (Spearman’s rho = 0.51; *P* < 0.0001) (Fig. 3).

## DISCUSSION

Environmental dust sampling on livestock farms and its analysis by PCR provide information on potential exposure of animals and humans to infection by pathogens such as *C. burnetii*. The technique is easy to perform and allows estimation of *C. burnetii* loads (14, 32). Furthermore, it is a useful screening tool prior to additional studies involving animal sampling. The inconvenience is the interpretation of the results, as *C. burnetii* spore-like SCV forms can remain at low levels for years in the environment and test PCR-positive even if they are not viable bacteria (9). On the other hand, seroprevalence data also help to infer the infection status of the flock. Here, we investigated whether the combination of serology and PCR analysis of environmental dust samples could be used to assess *C. burnetii* infection status in small ruminant farms.

Results showed a widespread distribution of *C. burnetii* DNA in environmental dust of small ruminant farms from the Balearic Islands. However, only 6% of them showed high loads of *Coxiella*, a rate that increased up to 22.1% when both high or moderate loads were considered. Interestingly, in farms with high or moderate loads of *C. burnetii* DNA, the mean within-flock percentage of seropositive animals against *C. burnetii* was also the highest (around 35%), significantly higher compared to flocks from farms with low loads or *C. burnetii* DNA absence. These results were supported by the significant correlation between serological and environmental analyses, indicating that both dust PCR and serology could be used for an initial screening of *C. burnetii* infection in a farm. However, although PCR also detects non-viable bacteria, PCR of environmental dust is initially preferable because it does not require the handling of animals for sample collection. On the other hand, detection of *C. burnetii* antibodies by serology may indicate a recent infection, but it may also reflect a past infection that the animals have already overcome, meaning they no longer shed *C. burnetii* into the environment. Therefore, it is necessary to carry out additional analyses and compile information on the flock’s abortion history. In this study, based on the results of the initial screening of dust samples, 22% of small-ruminant flocks were identified as suspect for *C. burnetti* infection and would require further animal samplings to assess the infection status of the flock. Analysis of animal samples collected at parturition, when infected animals shed high amounts of *C. burnetii* via birth products, uterine fluids, feces and milk (12, 13, 33), would be necessary to detect an active *C. burnetii* infection in these flocks. A high seroprevalence in yearlings would indicate a recent infection with *C. burnetii* (34, 35). Here, 16.1% of the flocks, where yearling and adults were examined, had a percentage of seropositive animals ≥ 30% in both age groups, suggesting that *C. burnetii* infection might be active in the flock. These results revealed the importance of sera sampling in both age groups as an approach to evaluate *C. burnetii* infection status in an animal population.

When abortions occur, seroprevalence rates in goats are usually higher than in sheep (36). In this study, goat herds showed a significantly higher seropositivity compared to sheep. However, no differences in positivity of dust samples were observed between the different flock types (sheep, goat, or mixed) even though goat farms showed the highest mean bacterial load (non-significant) in dust. A previous study reported a significant association between *C. burnetii* bacterial loads in dust and the occurrence of Q fever-associated abortions (14), but this association was not observed in the present study. Conversely, farms holding larger flocks showed a higher risk of being *C. burnetii* PCR-positive in environmental dust. This agrees with a previous study that also described a significant and positive correlation between *C. burnetii* environmental contamination and the number of breeding females in the farm (14). The purchase of animals has also been identified as a risk factor for *C. burnetii* infection (37, 38). Contact with other herds (communal grazing) has also been described as a contributing factor to the spread of *C. burnetii* between grazing flocks (39, 40). However, the results of this study do not indicate a significant association of *C. burnetii* positivity in environmental dust with the purchase of animals or the contact with livestock at grazing. It is interesting to note that contact with wildlife was associated with a lower risk of *C. burnetii* contamination. This could be explained by the absence in the Balearic Islands of the wild ungulates, such as deer and wild boar, known to be reservoirs of *C. burnetii* (4). However, the lagomorph species present (*Oryctolagus cuniculus* and *Lepus granatensis*) have been described as potential reservoirs of *C. burnetii* in Spain (41, 42). More research is needed to confirm the importance of the wild cycle of *C. burnetii* in the Balearic Islands. Among farm facility characteristics, poor ventilation was associated with an increased risk of *C. burnetii* contamination. Also, wearing clothing for exclusive use inside the animal facilities was shown to be a risk factor. This practice is associated with more professional farming systems that implement stricter biosecurity measures. However, professional farms also tend to have larger herds, which, as noted above, is in itself a risk for the presence of *C. burnetii* in dust.

*Coxiella* PCR-positive environmental dust samples can be subjected to strain genotyping. Here, we showed that SNP-6 is the predominant genotype in sheep and goat farms in the Balearic Islands. This genotype has been associated with the MST-8 genotype (43), detected in ruminants and humans in several European countries (Portugal, Switzerland, Italy, and France) and in North America (Canada and the USA) (22). In Spain, MST-8 has been detected in goats and cattle, as well as in humans (22). SNP-4 genotype was detected in a small number of flocks and has also been reported in northern Spain (15, 44). This genotype has been previously detected in bulk tank milk from goat farms in the Netherlands and in the blood of patients in Slovakia (26). Pearson and colleagues (43) inferred that SNP-4 could correspond to MST-20, a genotype mainly associated with cattle (43, 45, 46).

It is interesting to note the differences between the Balearic Islands and the northern part of Spain (Basque Country). In fact, in the province of Bizkaia (Basque Country), the predominant genotypes in small ruminants are SNP-8 and SNP-1 (15). Both genotypes have been related to human Q fever outbreaks involving pneumonia and with goats as potential origin of the outbreaks (11, 27–29). Conversely, SNP-6 has been detected less frequently in goats in the Basque Country (15). This differs from the epidemiological situation in the Balearic Islands, where, according to this study, SNP-6 is the predominant genotype in sheep and goat farms, suggesting that this genotype could be the origin of the clinical profile of human Q fever observed in the region, where pneumonia and hepatitis show comparable prevalence (30, 31).

The results of this study provided, for the first time, a global overview of the situation of *C. burnetii* in sheep and goats in the Balearic Islands and identified the factors associated with higher risk of environmental *C. burnetii* contamination. Strain characterization allowed the identification of the most prevalent *C. burnetii* genotypes in this region of eastern Spain, showing clear differences with the northern area, which could explain the different clinical spectrum of human cases of Q fever in both geographical areas. This study demonstrates the relevance of dust and sera sampling in ruminant farms to identify herds potentially infected with *C. burnetii* to further define priority actions and implement control measures. In addition, if combined with molecular genotyping and spatial distribution analysis (15), this strategy can help identify risk areas and trace the origin of human outbreaks (47).

## MATERIALS AND METHODS

### Study area and sampling

Between November 21, 2021, and September 30, 2022, in the context of the livestock Official Health campaigns in the Balearic Islands, dust samples were collected from inside the animal facilities of 249 farms (202 sheep, 18 goats, and 29 mixed [sheep and goats] flocks), representing 6.2% of the total number of small-ruminant flocks in the islands (ca. 4,000 flocks). In addition to domestic fauna (sheep, goats, cows, horses, donkeys, rabbits, dogs, and cats), 240 species of birds and several species of mammals of the families *Soricidae* (3), *Erinacidae* (1), *Chiroptera* (26), *Muridae* (5), *Gliridae* (2), *Leporidae* (2), *Mustelidae* (3), and *Viverridae* (1), and several species of reptiles are present in the islands.

Studied farms were randomly selected, and farmers gave written consent to participate in the study. Each farm was visited once to collect dust and sera samples. Sampling was carried out by the team of veterinarians participating in the official health campaigns. Prior to sampling, they received training on how to take dust samples to ensure homogeneous sampling. Whenever possible, at least 1 g of dust was collected from different surfaces of the facilities, preferably near the lambing areas, where dust accumulated (particularly windowsills and doors). Blood samples, collected during the official health campaign, were submitted to the laboratory within 24 h of collection, blood was centrifuged, and aliquots of 1.5 mL of serum were frozen until further serological tests.

The farmers answered a brief questionnaire with questions regarding the ruminant species present (type of flock, i.e., goats, sheep, mixed), breed, census, average lambing/kidding date, abortion problems (no/yes; year, if the answer was yes), management of fetuses and placentas, vaccinations, presence of ticks, contact with wildlife, recent purchase of animals, farming system (extensive, semi-intensive), contact with other herds at grazing, manure management (number of manure disposals per year), availability of clothing and footwear for exclusive use on the farm, reception of visitors and/or groups, farm characteristics (age, ventilation), use of straw bedding or steel grids, and origin of water.

### Molecular techniques

Dust samples (20–150 mg, mean 60 mg) were subjected to DNA extraction using a commercial kit (NZY Tissue gDNA Isolation Kit, NZYTech) and analyzed by real-time PCR targeting the multiple copy gene IS*1111* (48). A dust sample was considered *C. burnetii-*positive when the real-time PCR cycle threshold (Ct) was <40. Quantification of the bacterial load present in positive dust samples was performed by quantitative real-time PCR (qPCR) based on the detection of the single copy gene *com1* (49). Both PCR procedures were carried out as previously described (9). As proposed elsewhere (14, 15), bacterial loads (expressed in this study as genome equivalents, GE per mg of dust) were used to categorize the farms into four levels: (1) farms with high *C. burnetii* load (>1,000 GE/mg dust); (2) farms with moderate *C. burnetii* load (between 100 and 1,000 GE/mg dust); (3) farms with low *C. burnetii* load (<100 GE/mg dust); and (4) farms negative for the presence of *C. burnetii* DNA.

To investigate the *C. burnetii* genotypes present in the farms, a selection of DNAs with more than 40 GE/mg dust was analyzed by SNP genotyping following the protocol described elsewhere (26). Briefly, 10 real-time PCR reactions were performed per sample to detect 10 discriminatory SNPs, each including two primers and two MGB TaqMan probes to detect point mutations. Each 20 µL of PCR mixture contained 625 nM of each primer, 125 nM of each probe, 1 × Taq Mix ABsolute (Thermo Fisher Scientific), 10 µL of ABsolute QPCR Mix, no ROX (Thermo Fisher Scientific), 0.5 µL of bovine serum albumin (20 mg/mL) (New England Biolabs), and 4 µL of target DNA. PCR reactions were run on a QuantStudio 5 Real-Time PCR System (Thermo Fisher Scientific) under the following cycling conditions: 15 min at 95°C, and 40 cycles of 15 s at 95°C, and a minute at 60°C.

### Serological techniques

Based on the PCR results on dust samples, a maximum of 10 sheep, 10 mixed, and 10 goat farms were selected within each of the four farm categories defined based on the *C. burnetii* loads as described above. Sera were analyzed using a commercial ELISA kit (CHEKIT Q Fever Antibody ELISA kit, IDEXX, Liebefeld-Bern, Switzerland). This is an enzyme immunoassay with a mixture of phase I and II antigens of *C. burnetii* covering the microplate designed to be used on ruminant serum, plasma, and milk samples for the detection of IgG antibodies. In ruminants, as in humans, phase II antibodies have been associated with acute infection and bacterial clearance, whereas the production of phase I antibodies is a reflection of chronic infection (50). In principle, a kit with a mix of both phase I and II antigens would allow for the detection of both types of antibodies. The assays were performed according to the manufacturer’s instructions, grading the sera results as negative, doubtful, or positive based on the optical density obtained.

### Statistical analyses

Farms with incomplete questionnaires were excluded from the statistical analyses, leaving 223 farms with a complete data set. To statistically examine the possible associations of the real-time PCR (IS*1111*) result on dust (positive/negative) with the factors included in the questionnaire (detailed above), univariate, Chi-square (χ^2^), or Fisher analyses were performed. Only variables that showed significant differences in the univariate analysis were included in the multivariable logistic regression analysis. Thus, the following categorical variables were included: flock type (sheep, goats, and mixed flocks composed of sheep and goats), census (<50 heads, 50–100 heads, and >100 heads), contact with wildlife (yes/no), suffering from recent abortions (yes/no), exclusive use of specific clothing for animal facilities (yes/no), ventilation quality (good/poor), purchase of animals (yes/no), and use of straw bedding (yes/no). The best model was selected from all the models performed as the one with the lowest Akaike Information Criterion (AIC) value.

Chi-square (χ^2^) tests were performed (i) to compare the proportion of farms with different *C. burnetii* environmental contamination loads category (<100, 100–1,000, and >1,000 GE/mg dust) according to flock type and (ii) to assess differences in the proportion of seropositive animals between type of flock and age groups. Non-parametric Kruskal-Wallis test was used to compare (i) variations in *C. burnetii* environmental load (GE/mg dust) among flock types and (ii) differences in flock-level percentage of seropositive animals in relation to *C. burnetii* environmental contamination loads (<100, 100–1,000, > 1,000 GE/mg dust) and age (yearlings, more than one lambing/kidding). Where appropriate, Dunn’s test was applied following the Kruskal-Wallis test to identify pairwise differences among categories. To investigate the relationship between *C. burnetii* load (GE/mg dust) and within-flock percentage of seropositive animals, only dust-positive farms were selected, and Spearman’s rank correlation was used. The correlation coefficient (rho) and corresponding *P*-value were reported. Additionally, a simple linear regression line was superimposed on the scatter plot to visualize the overall trend. All statistical analyses were performed using R statistical software (51).
